# Excretion in the mother’s body: modifications of the larval excretory system in the viviparous dermapteran, *Arixenia esau*

**DOI:** 10.1007/s00709-018-1264-7

**Published:** 2018-06-09

**Authors:** Mariusz K. Jaglarz, Waclaw Tworzydlo, Szczepan M. Bilinski

**Affiliations:** Department of Developmental Biology and Invertebrate Morphology, Institute of Zoology and Biomedical Research, Jagiellonian University, Gronostajowa 9, 30-387 Krakow, Poland

**Keywords:** Malpighian tubules, Excretion, Spheroids, Viviparity, Dermaptera, Insects

## Abstract

The vast majority of Dermaptera are free-living and oviparous, i.e., females lay eggs within which embryonic development occurs until the larva hatches. In contrast, in the epizoic dermapteran *Arixenia esau*, eggs are retained within mother’s body and the embryos and first instar larvae develop inside her reproductive system. Such a reproductive strategy poses many physiological challenges for a mother, one of which is the removal of metabolic waste generated by the developing offspring. Here, we examine how the *Arixenia* females cope with this challenge by analyzing features of the developing larval excretory system. Our comparative analyses of the early and late first instar larvae revealed characteristic modifications in the cellular architecture of the Malpighian tubules, indicating that these organs are functional. The results of the electron probe microanalyses suggest additionally that the larval Malpighian tubules are mainly involved in maintaining ion homeostasis. We also found that the lumen of the larval alimentary track is occluded by a cellular diaphragm at the midgut-hindgut junction and that cells of the diaphragm accumulate metabolic compounds. Such an organization of the larval gut apparently prevents fouling of the mother’s organism with the offspring metabolic waste and therefore can be regarded as an adaptation for viviparity.

## Introduction

Insects show remarkable plasticity in adapting to various modes of life and to maximize their reproductive effort. Overwhelming majority of insect species lay eggs covered with hard protective envelopes and loaded with enough reserve materials (yolk) to sustain entire embryonic development (reviewed in Chapman 1998; Heming 2003; Wheeler 2003). In the oviparous mode of reproduction, maternal investment in progeny generally ends with the termination of oogenesis. However, a relatively small number of insect species employs a different reproductive strategy—viviparity. In viviparous insects, as in other animals with this mode of reproduction, mother commitment to offspring lasts much longer because oocytes/eggs are retained within the mother’s body and, following fertilization, developing embryos are nourished by the mother up to the time of larva hatching (Hagan 1951; Retnakaran and Percy 1985; Andrews and Rose 1994; Kalinka 2015; Ostrovsky et al. 2016). While processes associated with oviparity in insects are well characterized, little is known about morphological and physiological adaptations to viviparity in these arthropods.

Dermaptera is one of a handful of insect groups in which viviparity has been reported. Whereas most dermapterans are oviparous, species belonging to the Hemimeridae and Arixeniidae families are viviparous (Hagan 1951; Nakata and Maa 1974; Tworzydlo et al. 2013a). Because the viviparous dermapterans are epizoic, i.e., live non-parasitically on the body of certain mammals, it is believed that viviparity evolved to accelerate their life cycle and to ensure immediate contact of nymphs/larvae with an appropriate host (Hagan 1951; Nakata and Maa 1974). Our previous research revealed that in the viviparous dermapteran *Arixenia esau*, embryonic development is rather unusual and takes place both in a distal part of the ovariole, i.e., in the terminal ovarian follicle, and strongly dilated lateral oviducts or uteri (Tworzydlo et al. 2013a, b). In both compartments, the embryos are nourished by specialized maternal epithelial cells (Tworzydlo et al. 2013b). Providing sufficient nutrients for the embryos is just one of many challenges created by the viviparous reproductive strategy. Another vital challenge, for both the mother and developing inside her body embryos, is excretion. An intriguing question arises: How are the metabolic wastes, produced by offspring, eliminated from the reproductive system of their mother?

It is well established that in insects, regulation of intracellular homeostasis (excretion) is maintained by the Malpighian tubules and specialized regions of the alimentary canal (reviewed in Chapman 1998; Bradley 2003). The aim of this study was to explore morphological changes accompanying early development of the excretory organs in larvae of the viviparous dermapteran *Arixenia esau*, and how they relate to metabolic waste elimination during development inside the mother’s body.

## Material and methods

### Animals

The adult females of *Arixenia esau* Jordan, 1909 were collected from the walls of small caves (inhabited by bat colonies) in Bintulu District area, Sarawak, Malaysia. For this study, seven specimens were used. The number of larval instars in Arixeniidae has not been established yet; however, the complete absence of exuviae inside mother’s reproductive system indicates that all the larvae found inside the uteri should be classified as the first instar.

For the ease of description, the larvae dissected from the uteri were segregated into two developmental stages: early and late first instars, based on distinct morphological criteria. The early first instar larva does not exceed 5 mm in length. In contrast, the late first instar larva is larger (9–10 mm in length), covered with a thin and elastic cuticle with sclerotized setae and possesses a well-developed head with brownish pigmented compound eyes. These characteristics indicate that the late first instar larvae are almost ready for positioning.

The larval alimentary tracts with associated Malpighian tubules were dissected and fixed in a mixture of 2.5% glutaraldehyde and 1.5% formaldehyde in 0.1 M phosphate buffer, pH 7.4 for 5 days. Malpighian tubules from adult specimens were also processed as described above.

### Light and electron microscopy

The fixed samples were rinsed in 0.1 M phosphate buffer (pH 7.4) with sucrose (5.8 g/100 ml) and postfixed in 1% osmium tetroxide and 0.8% potassium ferrocyanide in phosphate buffer (pH 7.4) for 30 min at 4 °C. After dehydration in the graded series of ethanol (3 × 10 min in 30%, 50%, 70%, 90%, and 3 × 30 min in 100%) and acetone (3 × 10 min), the material was embedded in an epoxy resin Epon 812 (Serva, Heidelberg, Germany) according to the manufacturer’s protocol. Semi-thin sections (0.7–1 μm thick) were stained with 1% methylene blue and examined in a Nikon Eclipse Ni (Tokyo, Japan) or a Leica DMR light microscopes (LM) (Heidelberg, Germany). Ultrathin sections (80 nm thick) were contrasted with uranyl acetate and lead citrate according to standard protocols and analyzed with a transmission electron microscope (TEM) Jeol JEM 2100 (Tokyo, Japan) at 80 kV.

### Scanning electron microscopy

For the scanning electron microscopy (SEM), the material was fixed and postfixed as described above. After dehydration in graded series of ethanol, the material was critical-point dried in the Quorum Technologies E 3000 dryer (Lewes, UK), coated with gold in the sputter coater JEOL JFC 1100E (Tokyo, Japan) and examined with the Hitachi S-4700 (Tokyo, Japan) scanning electron microscope at 25 kV.

### Quantification of the elemental composition

For quantification of the elemental composition in the studied tissues, electron probe microanalyses were performed. Briefly, the resin sections attached to glass slides were coated with carbon and analyzed in the Hitachi S-4700 scanning electron microscope equipped with the energy dispersive spectrometry (EDS) analytical system (liquid-nitrogen cooled lithium drifted silicon (Si(Li)) x-ray detector and the Thermo Scientific NSS spectral imaging system). The standardless method was used for quantification of the elemental composition (for details see Trincavelli et al. 2014; Moy et al. 2015). For the control, analyses were performed on the tissue-free resin fragments of the same section.

## Results

**Fig. 1:**
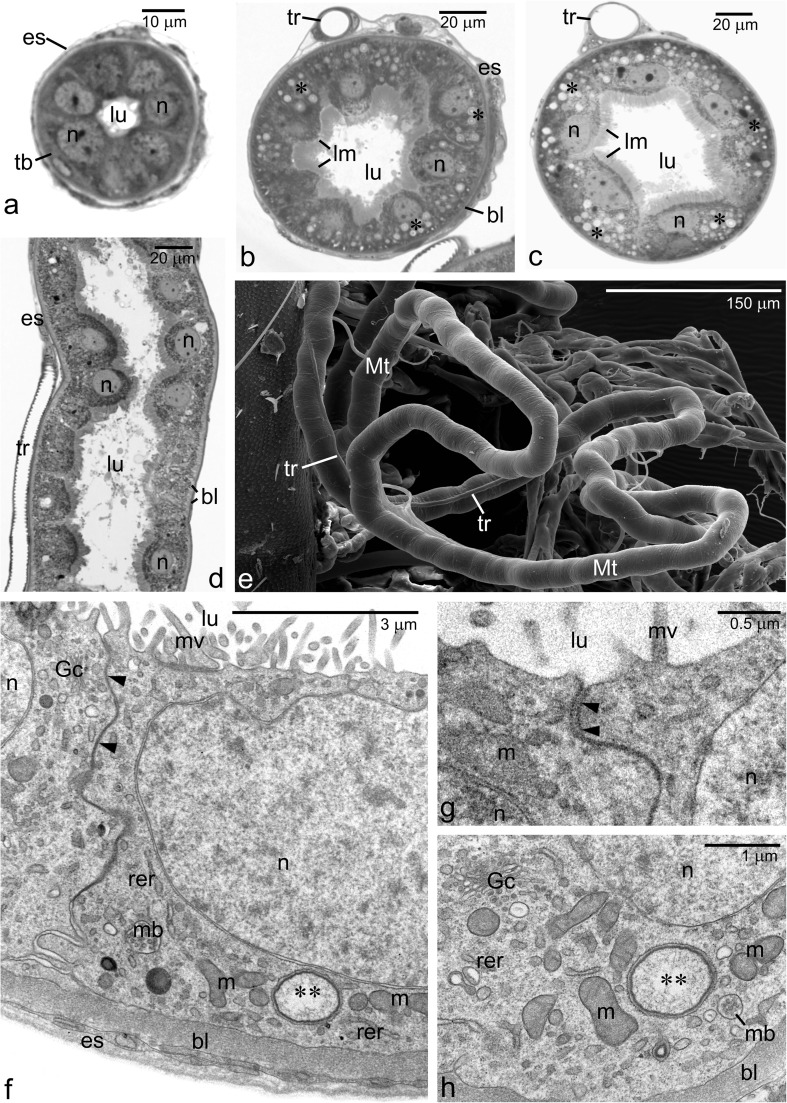
Malpighian tubule (Mt) development in *A*. *esau*. **a**–**c** Cross sections of Mt in the early first instar larva (**a**), late first instar larva (**b**), and adult (**c**). Note numerous spheroids in the Mt epithelial cells (**b**, **c**, asterisks). **d** Longitudinal section of Mt in the late first instar larva. **e** Scanning electron micrograph of Mts in the late first instar larva. **f**–**h** Ultrastructure of epithelial cells of the Mt in the early first instar larva. **f**, **g** Fragments of two neighboring Mt epithelial cells. Note a junctional specialization (arrowheads) between the adjacent membranes of the cells. Higher magnification reveals that these junctions are of the septate junction type (**g**, arrowheads). **h** The basal part of Mt epithelial cell. Note that the cell membrane is flat and runs parallel to the basal lamina (bl). *Asterisks* spheroids, *double asterisks* primordial spheroids, *bl* basal lamina, *es* epithelial sheath, *Gc* Golgi complex, *lm* layer of microvilli, *lu* Mt lumen, *m* mitochondria, *mb* multivesicular body, *mv* microvilli, *n* epithelial cell nucleus, *rer* elements of rough endoplasmic reticulum, *tb* tracheoblast, *tr* tracheole, **a**–**d** Semithin section stained with methylene blue, LM. **e** SEM. **f**–**h** TEM

To characterize the structure and functioning of excretory organs during development of juvenile forms of *Arixenia esau*, we have examined the cellular organization of Malpighian tubules (Mts) in the adults and the first instar larvae (segregated into early and late stages—see “Material and methods” section for detailed description) dissected from the mother’s uterus. To ease a comparison, the morphology of Mts in subsequent developmental stages is shown in Fig. 1a–e.

### Structure and ultrastructure of the Mts in the early first instar larva

The excretory organs of the early first larval instar consist of a dozen or so Malpighian tubules which arise from the intestine at the midgut-hindgut junction. The highly elongated, tangled, and blindly ending distal segments of the tubules lie freely in the hemocoel. The external diameter of Mts is about 40 μm, and narrows toward a blind tip of the tubule. The analysis of cross sections revealed that the tubules are morphologically complex and built of four layers (from inside out) (Fig. 1a, f): (1) a ring of cuboidal cells forming a single-layered epithelium lining a tubule lumen; (2) relatively thick mat of extracellular matrix forming a basal lamina which supports the epithelium; (3) a thin external sheath composed of striated muscle fibers and single tracheoblasts, the progenitor of tracheoles; and (4) a layer of basal lamina covering the external sheath.

The epithelial cells are morphologically similar and polarized along the apical-basal axis. Their apical plasma membranes, facing the tubule lumen, are furnished with relatively short (around 0.5 μm in length) and irregularly spaced microvilli (Fig. 1f). The lateral membranes run wavy but are tightly apposed to each other (Fig. 1f, g). They are held together by parallel rows of junctional structures arranged with a regular periodicity, features typical of a septate junction or septate desmosome (Fig. 1g). The lateral membranes close to the basal region (roughly up to a third of the cell height) are convoluted and separated by a narrow intercellular space (Fig. 1f). The basal membranes (facing the hemocoel) are flat and run parallel to the basal lamina (see below) to which are fastened by hemidesmosomes (not shown).

Each epithelial cell contains a large, ovoid nucleus which is clearly shifted toward the apical plasma membrane (Fig. 1a, f). The nuclear envelope is mostly regular except for the segment directed toward the cell apex where it deeply invaginates into the nucleoplasm. Each nucleus contains 2–3 prominent nucleoli and largely dispersed chromatin (Fig. 1a). The cytoplasm comprises numerous mitochondria, cisternae of the rough endoplasmic reticulum (RER), and Golgi complexes (Fig.1f–h). Single multivesicular bodies as well as characteristic spherical vacuoles reside preferentially in the basal region of the epithelial cells (Fig. 1f, h). The spherical vacuoles contain flocculent material of low or medium electron density, and electron dense material forming a peripheral rim (Fig. 1f, h). We interpret these vacuoles as an initial stage in the formation of spheroids (see the next paragraph).

### Structure and ultrastructure of the Mts in the late first instar larva (just before larviposition)

At this developmental stage, the diameter of the Mts increases considerably to around 110 μm (± 5 μm) and the internal lumen expands up to 40 μm (± 4 μm) in diameter (compare Fig. 1a, b). The analysis of Mts in cross sections revealed that the size increase is brought about by a significant enlargement of individual cells and their divisions. The average length of the cell apico-basal axis is 35 μm (versus 10 μm in the early first instar larva). In the late larva, cells of the Mt’s epithelial lining protrude more into the tubule lumen that gives the lumen a characteristic star-shaped appearance in cross sections (Fig. 1b). Within the lumen, numerous dense structures are often present (Fig. 1b, d).

**Fig. 2:**
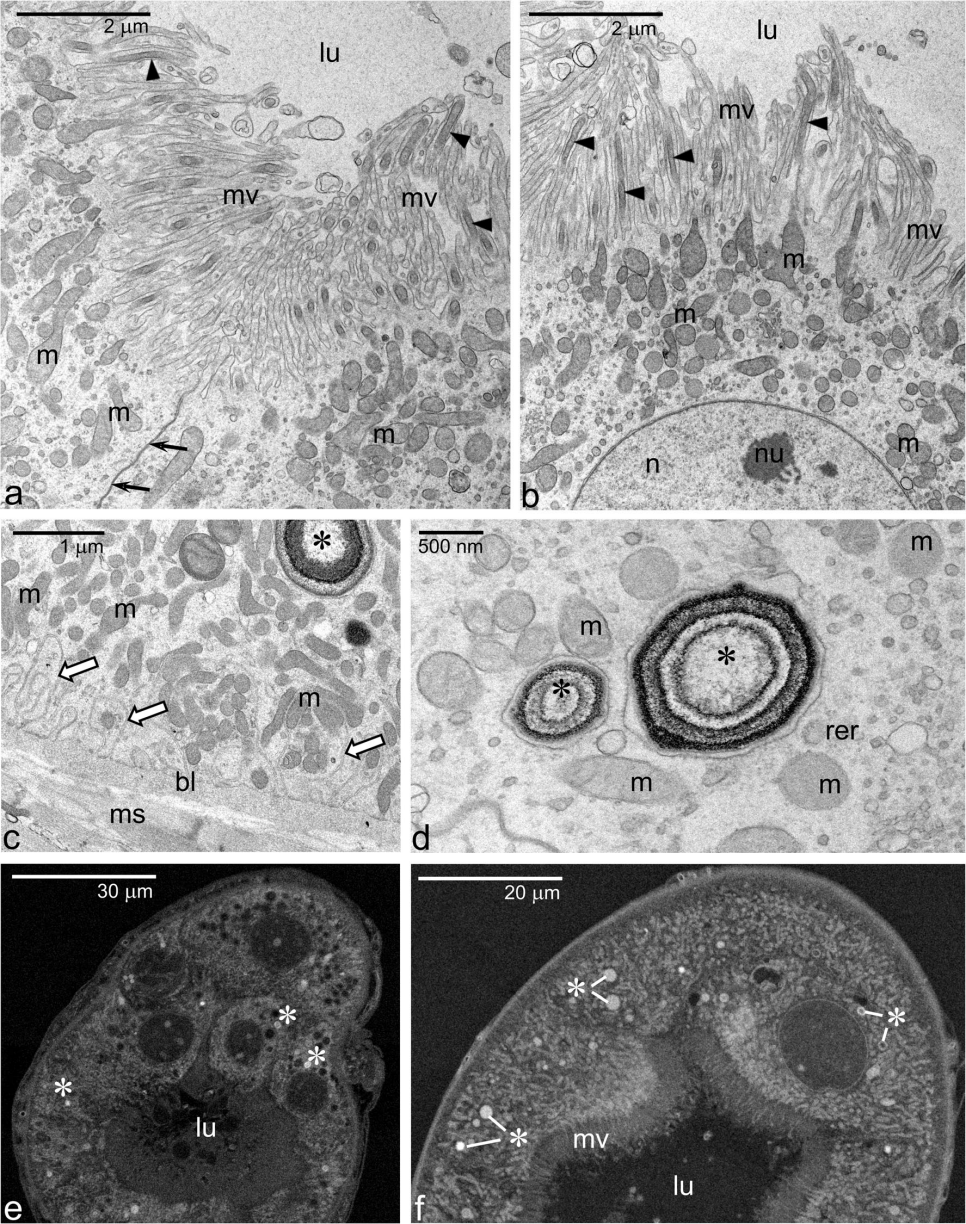
Malpighian tubule ultrastructure in the late first instar larva. **a**, **b** Apical regions of the Mt epithelial cells. Note numerous long microvilli (mv) containing elongated mitochondria (arrowheads). **c**, **d** Basal region of the Mt epithelial cells. Note that the basal membrane forms numerous infoldings (white thick arrows) containing mitochondria. The cytoplasm of Mt epithelial cells contains vesicles comprising spheroids (asterisks). **e**, **f** Representative SEM images of cross sectioned Mt in which elemental composition was analyzed. In such preparations, spheroids (asterisks) are clearly recognizable facilitating the electron probe microanalyses. *bl* basal lamina, *lu* Mt lumen, *m* mitochondria, *ms* muscle fibers, *mv* microvilli, *n* epithelial cell nucleus, *nu* nucleolus, *rer* rough endoplasmic reticulum arrows indicate intercellular junctions. **a**–**d** TEM. **e**–**f** SEM

Striking differences are also evident in the ultrastructural features of the epithelial cells of Mts when compared to the earlier developmental stage. First of all, the apical-basal polarity of the epithelial cells is even more pronounced. The apical surface, facing the lumen, is convex and extends into numerous slender and densely packed microvilli (Figs. 1b and 2a, b). The most characteristic feature of these microvilli is that each one of them contains a highly elongated mitochondrion, aligned parallel to a long axis of the microvillus (Fig. 2a, b). The cytoplasm just beneath the microvilli stains more intensely with methylene blue in semi-thin sections and the electron microscopy analysis revealed that this region of cytoplasm is densely packed with mitochondria, which form a crescent-shaped zone above the nucleus (Fig. 2a, b). Many mitochondria in this cytoplasmic region are irregular or elongated, oriented parallel to the long axis of the microvilli, and/or wedged into their base indicating transition steps necessary for entering the lumen of a microvillus (Fig. 2b). Similarly to Mts of the early larval instar, the apical regions of the neighboring cells are fastened by intercellular junctions (Fig. 2a). In contrast to the apical region, the basal cell membranes are extensively infolded (Fig. 2c). As a result, the basal cytoplasm is partitioned into irregular segments/compartments separated by narrow extracellular spaces. Typically, the cytoplasm of the basal segments is enriched in mitochondria (Fig. 2c).

There is no much difference in general structure and location of a nucleus in the epithelial cells of Mts in the early and late first instar larvae. However, the organization of the cytoplasm is markedly different. In the late first instar larva, the epithelial cells contain numerous, variably sized vesicles comprising spherical or ovoid granules with a distinct ultrastructural organization: concentrically arranged opaque and transparent zones (Fig. 2c, d). These granules will be referred to as the spheroids because of their morphological similarity to such structures described in Mts of other insects. The limiting membrane surrounding spheroids is covered with ribosomes (Fig. 2d) and in some sections is continued with membranes of RER elements (not shown). This observation suggests that spheroids are formed within expanded cisternae of RER. Although the spheroids are distributed throughout the cytoplasm, they are mostly concentrated in the basal half of the epithelial cells (Fig. 1b). In a single cell, the population of spheroids is highly heterogeneous with individual spheroids differing in size (from 100 nm up to 1.5 μm in diameter) and the extent of the concentric lamination (Fig. 2c, d). Serial section analyses have indicated that smaller vesicles contain material of lower electron density, more diffuse, and with a lower degree of lamination than the larger ones. Beside spheroids, multivesicular bodies are also present in the cytoplasm (not shown).

In the late first instar larva, each Mt is accompanied externally by a single unbranched tracheole which runs parallel to the long axis of the tubule (Fig. 1b, d, e). The analysis of the tracheoles in both cross and longitudinal sections revealed that they have a well-developed cuticular intima, which is annularly or helically folded to form a taenidium (Fig. 1d).

### Structure and ultrastructure of the Mts in the adult

We have also analyzed the structure of Mts in adult specimens of *A*. *esau*. We found that there is a great deal of similarity between the cell architecture of the late first larval instar and adult Mts, including their size and apico-basal polarity of the epithelial cells (compare Fig. 1b, c). The only noticeable ultrastructural differences are larger (up to 3 μm in diameter) and more numerous spheroids dispersed throughout the cytoplasm (not shown).

### Alimentary system

Although a detailed characteristic of the alimentary system in *A*. *esau* is beyond the scope of this study, here we present a brief description of the first instar larval gut only focusing on aspects relevant to the excretion and viviparity.

**Fig. 3:**
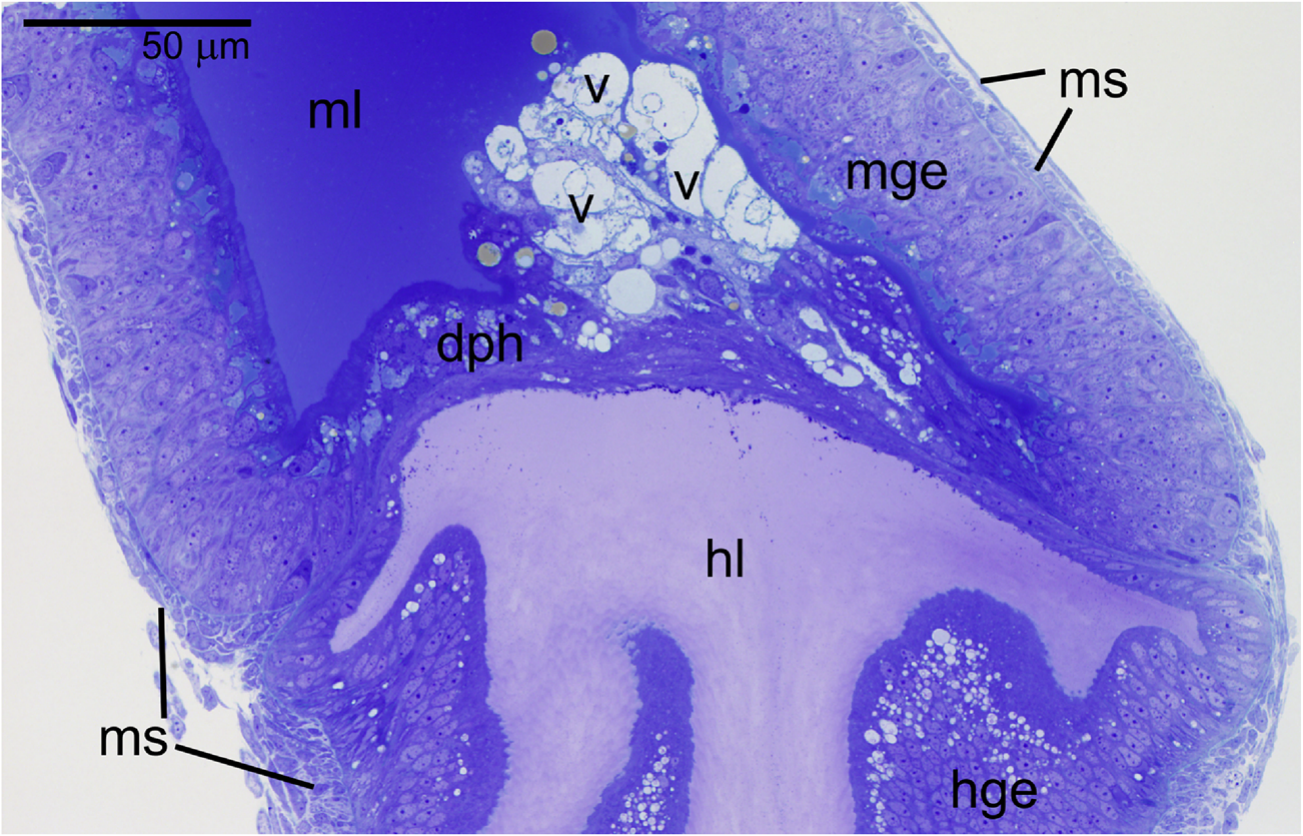
Longitudinal section of the fragment of the alimentary track in the late first instar larva. The continuity of the lumen is disrupted, at the midgut-hindgut junction, by a presence of the cellular diaphragm (dph) which is devoid of any muscle layers. Note large heterogeneous vacuoles (v) sticking out into the midgut lumen (ml) and a difference in staining between the midgut and hindgut lumens. *hl* hindgut lumen, *hge* hindgut epithelium, *mge* midgut epithelium, *ms* muscle fibers. LM

The alimentary systems of both the early and late first instar larvae are morphologically similar and comprise a foregut, midgut, and hindgut. In these larval stages, the most striking feature of the intestinal tract is occlusion of its lumen by a cellular diaphragm at the midgut-hindgut junction (Fig. 3). The serial section analysis revealed that the diaphragm is a continuous solid cellular layer and is formed by adjacent blind ends of the midgut and hindgut tubes. Significantly, there is no muscle lining associated with this structure (Fig. 3). The muscle layers form a sheath surrounding externally both the midgut and hindgut but they do not penetrate into the diaphragm region. Such an arrangement allows to hold the gut parts together even though their cavities remain separated (Fig. 3). Another indication of the gut discontinuity is the observed disparity in the staining properties of the midgut and hindgut lumens (Fig. 3), confirming that the diaphragm forms a tight diffusion barrier between these two gut compartments.

**Fig. 4:**
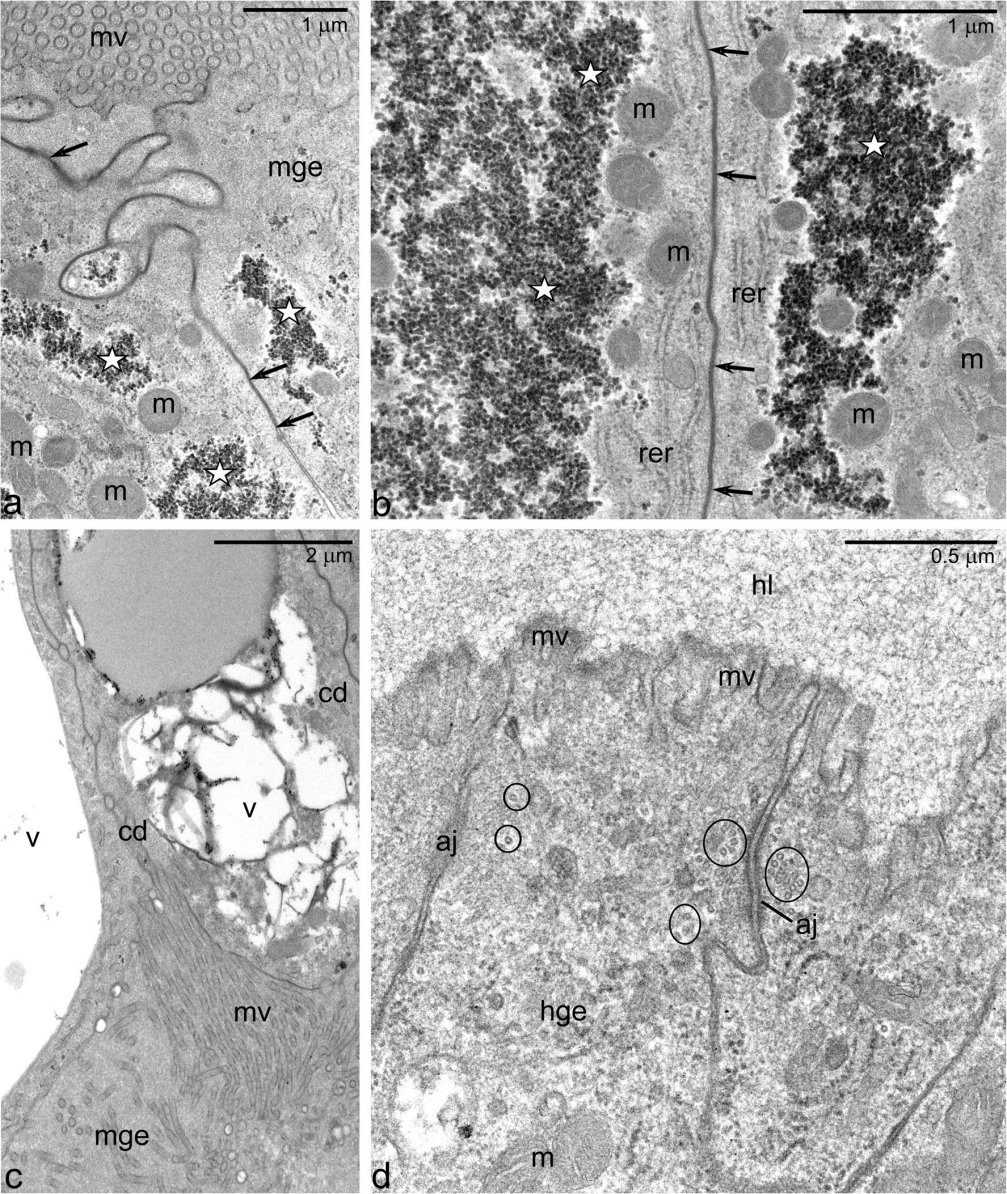
Ultrastructure of the larval gut. **a**, **b** Neighboring midgut epithelial cells (mge) are fastened with continuous junctions (arrows). Apart from mitochondria (m) and elements of rough endoplasmic reticulum (rer), these cells contain large accumulations of glycogen-like particles (white stars). **c** Heterogeneous vacuoles (v) in the cell of the diaphragm (cd). **d** The apices of the hindgut epithelial cells (hge) are furnished with very short microvilli (mv) covered with a delicate layer of the cuticle. The lateral membranes in the apical regions are linked by the adherens junctions (aj) accompanied on either side by microtubules (encircled). *hl* hindgut lumen, m mitochondria, *mge* midgut epithelial cells,* mv* microvilli. TEM

In the *Arixenia* first instar larvae, a clear morphological distinction exists between midgut and hindgut cells. The wall of the midgut is relatively thick (approximately 50 μm in diameter) and consists of a thin outer muscular layer and a well-developed inner epithelial lining of the gut (Fig. 3). The apical membranes of the midgut epithelial cells (exposed to the gut lumen) are furnished with multiple microvilli (about 100 nm in diameter and more than 5 μm in length) (Fig. 4a), whereas the lateral ones are connected by extensive continuous junctions, or *zonulae continuae* (Fig. 4a, b). The cytoplasm of these cells contains irregular aggregates of electron dense granules (Fig. 4a, b). Both the shape and size of these granules suggest that they may represent glycogen particles. Similar aggregates are also present in the endodermal cells of the diaphragm (facing the midgut lumen), where they intermingle with large vacuoles filled with a heterogeneous content, which will be referred to as the heterogeneous vacuoles (Figs. 3 and 4c). The aggregates and vacuoles present in the diaphragm cells often form massive accumulations filling most of the cell’s interior. Some cells of the diaphragm are distended and distorted by these accumulations and stick out into the otherwise empty midgut cavity (Fig. 3).

The lining of the hindgut is organized in a single-layered epithelium. The hindgut epithelial cells are highly elongated or columnar (Figs. 3 and 4d); in contrast to midgut cells, their apices form only a small number of short microvilli (about 70 nm in diameter and 250 nm in length) (Fig. 4d). Characteristically, the apices of these microvilli are covered by a delicate layer of cuticle (Fig. 4d). The apical domains of the hindgut epithelial cells are fastened by the adherens junctions, which are accompanied on either side by microtubules (Fig. 4d).

### Electron probe microanalyses

**Fig. 5:**
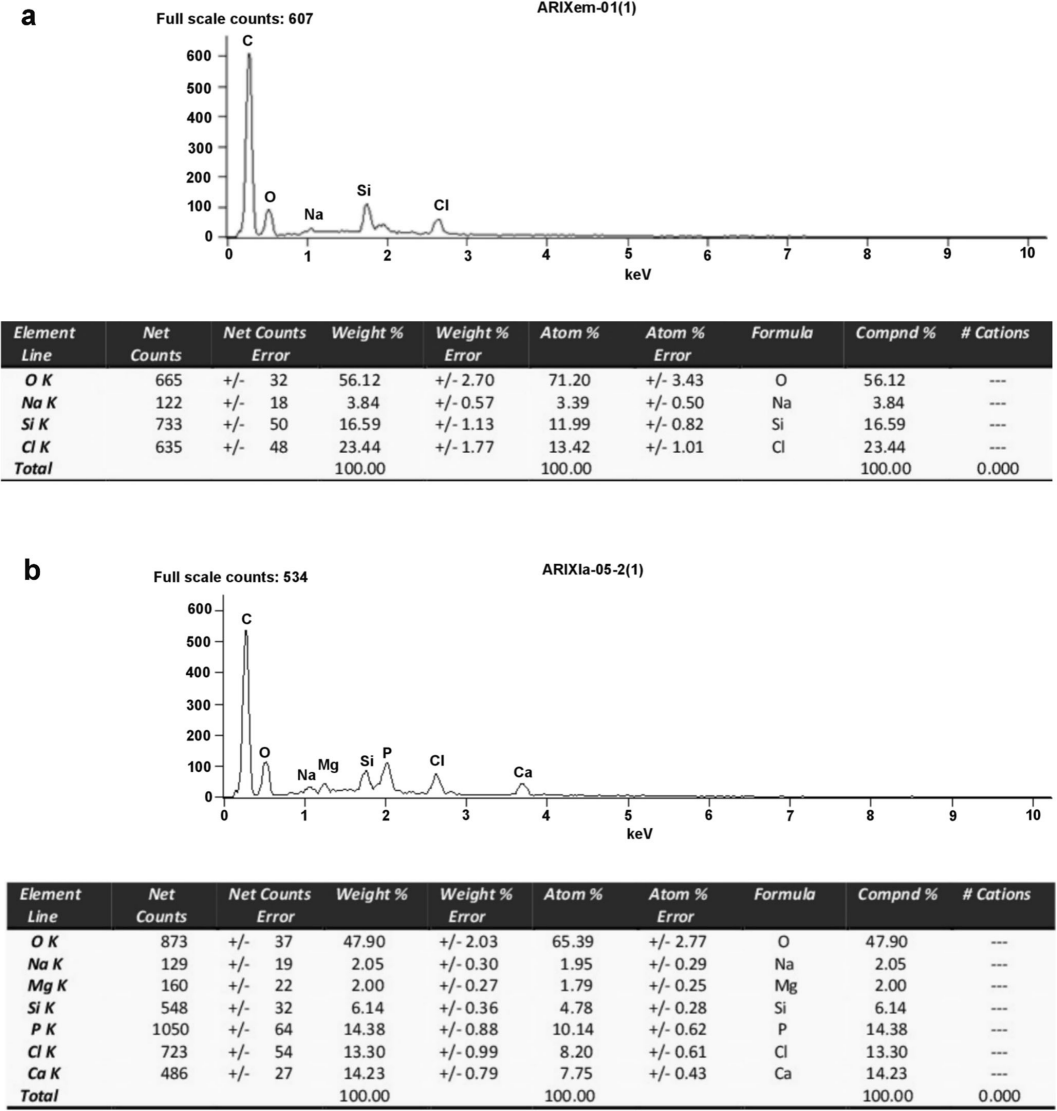
Representative EDS spectra of Mt organelles. **a** Vacuoles in the early first instar larvae. **b** Spheroids in the late first instar larva (like the ones marked with white asterisks in Fig. 2f). The accompanying tables show the elemental composition (compound %) of these organelles

To better characterize vacuoles and spheroids present in the epithelial cells of the Mt and midgut and to determine their chemical composition, we have analyzed their contents with the EDS system. Exemplary images of the Mt sections used for the microanalyses are shown in Fig. 2e, f. These analyses revealed that the vacuoles found in the Mt epithelial cells of the early first instar larvae contain no measurable amount of elements such as nitrogen, calcium, or phosphorus (Fig. 5a). In contrast, the spheroids in the late instar larval Mts contain variable but significant amount of calcium and phosphorus, i.e., around 14 wt% (Fig. 5b). These amounts were well above the content of these elements in the cytoplasm surrounding spheroids and on a tissue-free area of the same section (not shown). The obtained calcium/phosphorus ratio close to 1 suggests that these elements may form compounds, e.g., calcium phosphate, within the spheroids.

We have also analyzed the composition of the heterogeneous vacuoles present in cells of the gut diaphragm and found that at least some of them have a slightly elevated amount of calcium (2.5 ± 0.44 wt%).

## Discussion

As in other animals, excretion in insects is necessary to maintain proper intracellular environment and homeostasis of the entire organism. Excretion relays on the elimination of metabolic waste, excess water and ions from the hemolymph, which is the reservoir of the extracellular fluids (reviewed in Bradley 2003). Metabolic waste produced during various cellular activities is transported into the hemolymph and removed from it primarily by Mts (for reviews, see Pannabecker 1995; Bradley 1998; Beyenbach et al. 2010). In viviparous mode of reproduction, several excretory scenarios are possible, including a total dependence of the progeny on the excretory system of the mother (as it is in hemocelic viviparity), a partial contribution on mother’s side, or a total excretory independence of progeny (Clutton-Brock 1991; Blackburn 1999). Our results indicate that the last scenario is likely to operate in the studied viviparous dermapteran. The excretory system develops in concert with the overall development of the *Arixenia* larva and appears to act independently of the mother’s excretory system.

The ultrastructural analysis of Mts in two successive developmental stages revealed that the epithelial cells gradually acquire distinct apico-basal polarity, increase in size, and progressively differentiate into a secretory epithelium. These results combined with the expansion of the lumen of Mts and the rising number of vesicles containing spheroids within the cytoplasm of the Mt epithelial cells strongly indicate that the larval Mts become gradually functional during *in utero* development.

The presence of spheroids, also termed urospheroids, spherocrystals, concentric concretions, and crystalloid bodies, are of special interest. These structures are thought to be depots of insoluble form of salts containing different ions (calcium, copper, magnesium, cadmium, manganese, and zinc) embedded in organic, mostly proteinaceous matrix (reviewed in Martoja and Ballan-Dufrançais, 1984; Chapman 1985; Bradley 2003). Although, the exact role of the mineralized concretions may depend on a species, it has been suggested that they store the ions either for subsequent use by the organism or as a means of sequestrating and eliminating of ions from the body. Elements of RER are usually implicated in the origin of spheroids, which is in line with our findings. There is very little variability in mature spheroids among insect species: they are always roughly spherical and have a characteristic concentric pattern of transparent and opaque rings (for reviews, see Martoja and Ballan-Dufrançais, 1984; Chapman 1985). This may suggest that similar mechanisms are responsible for their formation throughout Hexapoda.

Morphologically similar structures to spheroids have been also reported in midgut cells in various hexapods (e.g., Humbert 1979; Chapman 1985; Xue et al. 1990; Pigino et al. 2005; Rost-Roszkowska et al. 2007; Rost-Roszkowska and Undrul 2008). Although we have not observed canonical spheroids in the midgut cells of *Arixenia*, we have found large heterogeneous vacuoles in their cytoplasm. The electron probe microanalysis revealed the presence of calcium in at least some of these structures. Calcium, in turn, is considered to be the most typical and ubiquitous constituents of the spheroid-like structures (Chapman 1985).

Several lines of evidence indicate that insect Mts are involved in primary urine formation (for reviews, see Martoja and Ballan-Dufrançais, 1984; Bradley 1985, 2003; Pannabecker 1995; Beyenbach et al. 2010). We believe that *Arixenia* larval Mts participate in the same process. This notion is supported by remarkable similarity of the ultrastructure of the *Arixenia* larval Mt epithelial cells to the ultrastructural organization of such cells in many insects (e.g., Ryerse 1979; Hanrahan and Nicolson 1987; Arab and Caetano 2002; Goncalves et al. 2014; Lipovsek et al. 2017; Liu and Hua 2018). It should be mentioned here that in larvae of some holometabolous insects, e.g., *Apis mellifera*, differences in Mt organization may occur that are associated with substantial remodeling of the internal organs accompanying complete metamorphosis (for further details, see Goncalves et al. 2018).

In *Arixenia*, both the apical and basal surface modifications (microvilli and basal infoldings, respectively) greatly increase the effective transport area of the Mts across which metabolites may pass between the hemolymph and cytoplasm and between the cytoplasm and the excretory space. The septate junctions between the cell lateral membranes provide structural strength as well as a barrier to ion and solute diffusion through the intercellular space (Lane 1982). The observed juxtaposition of mitochondria and the cell membranes, either in the form of the apical microvilli or basal infoldings, suggests the presence of an active transport mechanism, requiring energy provided by the abundant mitochondria. Such ultrastructural organization is characteristic of transporting epithelia involved in osmotic and ionic regulation (Berridge and Oschman, 1972). Similar associations of mitochondria with microvilli-like projections have also been reported in goblet cells, present in the midgut of some insects (e.g., Ephemeroptera, Lepidoptera), and implicated in the regulation of potassium titers within the hemolymph (reviewed in Martoja and Ballan-Dufrançais, 1984; Chapman 1985).

Additionally, the results obtained with the EDS system indicate that Mts of the early and late larvae differ in concentration of calcium and phosphorus. These results strongly suggest that as the *Arixenia* first instar larva develops, the Mts become progressively more involved in regulation and elimination of excess ions and water. The surplus of ions is apparently sequestered (probably in the form of calcium phosphates) in the spheroids gradually arising in the cytoplasm of the Mt epithelial cells. Our results suggest additionally that the epithelial cells forming the diaphragm may support the excretion. We speculate that heterogeneous vacuoles present in these cells may act as storage depots for wasteful byproducts of metabolism generated by a developing in the mother’s uterus larva. This hypothesis is reinforced by the observed higher calcium content in these vacuoles.

Our microscopic analyses indicate that the gut in the *Arixenia* first instar larvae is discontinuous, does not function in the canonical way, and that the epithelial cells of the diaphragm accumulate metabolic compounds. We interpret such unusual modification of the alimentary system as an adaptation for viviparity. Because the larva develops inside the mother’s uterus, the occlusion of the gut lumen may prevent polluting the environment (i.e., the uterus lumen) with the metabolic waste. We believe that the gut becomes patent after larva deposition either as a result of rupturing the cellular diaphragm by passing food or at the first molt.

In many insects, the midgut/hindgut junction is equipped with pyloric valves. However, several lines of evidence indicate that the diaphragm in the *Arixenia* first instar larvae is a different structure. First, the pyloric valves are always associated with a thick muscle layers (see Chapman 1985 for further discussion), while the diaphragm is completely devoid of musculature. Second, in contrast to pyloric valves, the diaphragm is a solid cellular layer, as indicated by a serial section analysis. Third, different staining properties of the midgut and hindgut lumens argue that the diaphragm provides a tight diffusion barrier, which would not be the case with the pyloric valves.

Interestingly, an occlusion at the midgut/hindgut junction has been also reported in larvae of neuropterans, most hymenopterans and viviparous dipterans (reviewed in Chapman 1985). At least in the case of viviparous dipterans and social hymenopterans, the gut discontinuity may play a similar role as suggested for *Arixenia*.
